# Noncanonical Ion Channel Behaviour in Pain

**DOI:** 10.3390/ijms20184572

**Published:** 2019-09-15

**Authors:** Cosmin I. Ciotu, Christoforos Tsantoulas, Jannis Meents, Angelika Lampert, Stephen B. McMahon, Andreas Ludwig, Michael J.M. Fischer

**Affiliations:** 1 Center for Physiology and Pharmacology, Medical University of Vienna, 1090 Vienna, Austria; 2 Wolfson Centre for Age-Related Diseases, King’s College London, London SE1 1UR, UK; 3 Institute of Physiology, University Hospital RWTH Aachen, 52074 Aachen, Germany; 4 Institute of Experimental and Clinical Pharmacology and Toxicology, Friedrich-Alexander-Universität Erlangen-Nürnberg, 91054 Erlangen, Germany

**Keywords:** pharmacology, drug development, sodium channel, potassium channel, TRP channel, HCN channel

## Abstract

Ion channels contribute fundamental properties to cell membranes. Although highly diverse in conductivity, structure, location, and function, many of them can be regulated by common mechanisms, such as voltage or (de-)phosphorylation. Primarily considering ion channels involved in the nociceptive system, this review covers more novel and less known features. Accordingly, we outline noncanonical operation of voltage-gated sodium, potassium, transient receptor potential (TRP), and hyperpolarization-activated cyclic nucleotide (HCN)-gated channels. Noncanonical features discussed include properties as a memory for prior voltage and chemical exposure, alternative ion conduction pathways, cluster formation, and silent subunits. Complementary to this main focus, the intention is also to transfer knowledge between fields, which become inevitably more separate due to their size.

## 1. Overview

The main aim of this review is to illustrate unexpected behaviour of ion channels, which might cross-pollinate advances between fields. To at least partly fulfil this aim, we restricted coverage to the pain field and the subsections are written by authors with a focus on the respective ion channel families. We consider as canonical any feature of an ion channel pore-forming protein, which can allow the flow of ions across membranes [1]. Although not universal, common features include regulation of the permeation (gating) by ligands and voltage; preference or selectivity for some ions over others; interaction with other cytoplasmic or membrane proteins; trafficking between the plasma membrane and reserve pools; heteromerisation of the channels; modulation by intracellular cascades e.g., by a change of phosphorylation state; and a change of expression levels, e.g., in inflammatory conditions. Less common and more on the line between canonical and noncanonical are features such as interaction with phospholipids or accessory subunits.

## 2. Sodium Channels

Voltage-gated sodium channels (Navs) are responsible for the generation of action potentials in most excitable cells, such as neurons and muscle cells. Ten different isoforms have been described in mammals (Nav1.1-1.9 and Nax), which vary in tissue expression and electrophysiological properties [2,3]. Generally, Navs are highly voltage sensitive and open in response to small membrane depolarisations. They are selective for the conduction of sodium ions, thus amplifying membrane depolarisation and initiating action potentials. For the study and treatment of pain, the subtypes Nav1.7, Nav1.8, and Nav1.9, mostly expressed in peripheral sensory neurons, have received large interest over recent years considering that mutations in these channel isoforms can lead to a variety of pain syndromes in patients [4,5]. Well-described canonical features of Nav channel activity comprise voltage-dependent gating and fast inactivation during membrane depolarisation as well as channel deactivation upon cell membrane repolarisation [2,6,7]. Of note in this respect are the recently published 3-D crystal structures of different Nav isoforms that have shed a new light on these well-known functions [8,9,10,11]. There are, however, a number of rather unexpected and less well-understood channel functions that will be discussed in the following. Some of these have already been reviewed in a similar context by Barbosa and Cummins [12].

In addition to fast inactivation, which occurs within milliseconds after channel opening, Navs can also undergo slow inactivation, a process that takes place on a time scale of seconds to minutes. Under experimental conditions, this process can be observed during prolonged depolarisations (e.g., 30–60 s). Physiologically, slow inactivation is believed to take place during high-frequency firing, also serving to modulate it. Slow inactivation in Navs has been known for several decades [13,14]. Slow inactivation is different depending on Nav channel isoform, and the exact molecular determinants for slow inactivation are difficult to pinpoint as many positions and residues have been described that seem to affect slow inactivation. Generally, the process of slow inactivation in Navs bears similarities to the C-type inactivation of potassium channels and involves the channel pore [15]. Especially a ring of four negatively charged amino acids directly above the selectivity filter (E409, E764, D1248, and D1539 in hNav1.4 [9]) seems to be involved in this process [16]. However, many other residues both inside and outside the channel pore have been implicated in slow inactivation (reviewed in References [13,14]). With regards to nociception, slow inactivation has been found to be modulated by different mutations in Nav1.7 that cause the chronic pain syndrome erythromelalgia [17,18,19,20,21,22,23,24,25,26,27,28,29] (reviewed in References [12,30]). Slow inactivation in peripheral Navs seems to be enhanced by cold temperatures, with the exception of Nav1.8, which inactivates cold-independently and thus mediates cold nociception in mice [31].

Slow inactivation can be regarded as a sort of negative hysteresis, i.e., Nav channels “remember” a previous prolonged or high-frequency stimulation and, as a result, remain inactive. In other ion channels, such as transient receptor potential and potassium channels, different forms of hysteresis have been described, including an *increase* in conduction or sensitivity upon prolonged or repeated stimulation (see below and in Reference [32]. However, our group has failed to show changes in voltage dependence of activation of Nav1.7 after a series of depolarizing pre-pulses, thus questioning the role of positive hysteresis in Nav1.7 [33].

According to the canonical view, Nav channels inactivate milliseconds after opening and remain impassive to sodium flux until the cell membrane has repolarised and the channel has returned to its resting state. However, an unusual Nav current, termed resurgent current, has been described to occur during membrane repolarisation. Since their first description in the late ‘90s in cerebellar Purkinje neurons [34], resurgent currents have become highly investigated and are believed to modulate high-frequency action-potential firing in different types of neurons, including nociceptors [35]. The molecular process of resurgent currents consists of an open channel block by a positively charged intracellular blocking particle, which occludes the channel pore before fast inactivation occurs. During membrane repolarisation, this particle is released from the channel pore due to its positive charge, thus leading to a very brief inward sodium current before the channel inactivates [35,36]. The most likely candidate for the open channel blocking particle is the C-terminal end of the Navβ4 subunit [35,37]. Peripheral sensory neurons express fast and slow resurgent currents, mainly mediated by Nav1.6, Nav1.2, and potentially Nav1.8 [38,39,40]. Nav1.7 has also been shown to produce resurgent currents of small amplitudes. However, mutations in Nav1.7 that cause the chronic pain phenotype paroxysmal extreme pain disorder (PEPD) enhance resurgent currents in this Nav subtype, whereas mutations leading to erythromelalgia do not [29,41]. Interestingly, there seems to be a direct correlation between resurgent current generation and slow inactivation in Nav1.6 and Nav1.7: enhanced slow inactivation impairs resurgent currents and vice-versa [29].

Local anaesthetics can be a useful tool for quick and localized pain treatment. These drugs have been shown to bind inside the central cavity of the channel pore [42,43,44]. Recent findings in prokaryotic and mammalian Nav channels have substantiated earlier reports, which suggested that entry of local anaesthetics into the central cavity can be mediated via the lipid phase of the cell membrane [45]. The recently published 3-D crystal structures as well as earlier models show side fenestrations of the channel pore, which are large enough to be permeated by small molecules, such as local anaesthetics [9,10,46,47,48,49]. This may have important implications for the future development of Nav channel blocking compounds.

Several naturally occurring mutations in Nav1.2, Nav1.4, and Nav1.5 have been reported to conduct so-called gating pore (or omega) currents. These currents originate from mutations of gating charge residues in the S4 voltage sensor, leading to an alternative ion permeation pathway across the membrane [50,51,52,53,54] (reviewed in Reference [55]). Whereas such gating pore currents have not yet, to our knowledge, been investigated in nociceptive Nav channel isoforms, it might still be worthwhile to check for such currents, especially in Nav1.6–Nav1.9, as leak currents through these channels would almost certainly affect nociceptor excitability and pain perception.

## 3. Potassium Channels

Potassium channels are the most populous, diverse, and widely distributed ion channel superfamily. Once regarded as “innocent bystanders” that could nevertheless be pharmacologically exploited to counteract neuronal hyperexcitability rising from maladaptive activity of other ion channels, potassium channels are increasingly viewed as key players that can directly promote pain pathogenesis [56]. Indeed, an ever-growing number of studies report causative links between reduced function of specific potassium channel subunits and development of neuronal hyperexcitability and pain sensation [57]. Furthermore, in not electrically excitable cells, potassium channels participate in several neurophysiological processes that are independent of ion conduction, such as proliferation, migration, and exocytosis, and the mechanisms governing these noncanonical functions may also be of relevance to pain syndromes [58,59,60].

The best studied group, voltage-gated potassium channels (Kv), comprises 40 members which assemble as homo- or hetero-tetramers and mediate a hyperpolarising K^+^ efflux that limits neuronal excitability by opposing action-potential generation. Membrane depolarisation triggers Kv opening via movement of the voltage sensor, which is coupled through a 15aa helical S4-S5 linker to the channel pore [61]. Recent work, however, in Drosophila’s Shaker potassium channels (closely related to human Kv1 channels) identified an additional electromechanical coupling between residues of transmembrane domains S4 and S5 which facilitate movement of the helices in a “rack-and-pinion” fashion [62]. This noncanonical mechanism is a good candidate to explain pore opening in channels like hERG (Kv11.1), which contain a short S4–S5 linker, expendable for voltage-gating [63,64].

The traditional view of Kv opening exclusively gated by voltage was challenged by Hao et al., who demonstrated that Kv1.1 is a bona fide mechanoreceptor in sensory neurons [65]. In thorough experiments, it was shown that a variety of mechanical manipulations such as piezo-electrically driven force, membrane stretching, and hypoosmotic shock directly activate Kv1.1 channels to mediate a mechanosusceptive current, dubbed I_Kmech_. Mechanistically, generation of I_Kmech_ results from a change in the voltage dependence of the open probability, favouring the open conformation of the channel. Traditional mechanotransducers use a mechanical sensor linked to cytoskeletal elements to convert membrane tension energy into conformation changes. In contrast, Kv1.1 mechanoactivation may depend on inherent properties of the voltage sensor because mechanosensitivity is retained in excised patches of DRG neurons [65]. The authors postulated that the local membrane distortion induced by applied forces alters the energetic stability of the voltage-sensing machinery by physical movement of charges within the channel. Whatever the precise mechanism, Kv1.1 activation by mechanical stimulation can—because it reduces neuronal excitability—tune sensory neuron excitability by opposing excitatory influences of mechanosensitive cation channels. The net outcome of this process depends on the exact ionic channel complement of the neuron; in C-high threshold mechanoreceptors which mediate slowly adapting mechanosensitive cation currents [66], I_Kmech_ opposes depolarisation and increases mechanical thresholds. In contrast, in Aβ mechanoreceptors which encode rapidly adapting mechanosensitive currents, I_Kmech_ is not engaged sufficiently to influence firing thresholds but can nevertheless regulate firing rates. This elaborate control of mechanosensitivity by a Kv channel is a novel mechanism of mechanosensation, and inhibition of this pathway due to injury or inflammation could promote mechanically induced pain. Consistent with this, blocking Kv1.1 activity in mice either genetically or pharmacologically triggers mechanical hypersensitivity, without affecting heat pain responses [65].

The closely related member Kv1.2 also stands out because its function is subject to epigenetic silencing by G9a (histone-lysine N-methyltransferase 2) [67]. Neuropathic injury induces the Myeloid Zinc Finger 1 transcription factor, which in turn upregulates a long noncoding antisense RNA which attenuates Kv1.2 expression and activity, leading to hyperexcitability and pain sensitivity in rodents. Blocking induction of the antisense RNA spares Kv1.2 expression and is protective against pain [68]. This and other emerging pathways regulating Kv-dependent excitability via epigenetic modifications [69] might constitute a dynamic mechanism which shapes neuronal activity in development and disease [70,71]; the applicability of this theme in pain pathology remains to be further established but could critically inform gene therapy approaches in the near future.

Kv2.1 is another interesting channel as it features unique subcellular localisation, regulation by silent subunits, and nonconducting functions. Being a high-threshold channel with characteristically slow kinetics, Kv2.1 becomes particularly important during prolonged stimulation, like that encountered in central neurons during seizures [72,73], or in peripheral nociceptors during spontaneous firing. Accordingly, inhibiting Kv2.1 currents in DRG neurons allows higher firing rates during sustained input [74], while Kv2.1 knockout in the CNS results in neuronal hyperexcitability reminiscent of epilepsy [72]. It therefore appears that Kv2.1 acts as a resistor that filters elevated neuronal firing and is compromised in syndromes linked to neuronal hyperexcitability, including chronic pain. Kv2.1 is downregulated in damaged sensory neurons thus promoting hyperexcitability [74], but as Kv2.1 levels are not completely abolished, this may be exploitable for pharmacological enhancement with Kv openers. Constituent reduction of Kv2.1 activity can also occur via mutations in the *KNCB1* gene; missense variants located within the pore domain result in loss of K^+^ selectivity and generation of a depolarizing inward sodium current at negative voltages [75] or even loss of voltage dependence, causing Kv2.1 to remain tonically open [73]. It will be interesting to investigate whether similar Kv2.1 mutations are linked to human pain channelopathies.

Kv2.1 is robustly regulated by members of the Kv5, Kv6, Kv8, and Kv9 families, which comprise the so-called “silent subunits” (KvS). These enigmatic proteins are incapable of conducting currents on their own but can form functional tetramers with Kv2.1, substantially altering the biophysical properties of the channel [76]. For instance, association of Kv2.1 with any of Kv5.1, Kv6.1, Kv9.1, or Kv9.3 hyperpolarises the voltage dependence of inactivation in pyramidal neurons, while Kv2.1/Kv5.1 exhibits accelerated rates of (open-state) inactivation and slower closing rates upon repolarization (deactivation) [77]. Mechanistically, these modulatory effects can be mediated by direct changes in the gating mechanism or indirectly by promoting Ca^2+^/calmodulin-dependent dephosphorylation [77,78]. It is becoming increasingly evident that heteromerization of Kv2.1 with different KvS endows neurons with functional diversity that is often essential for normal physiology. For example, mammalian photoreceptors depend on Kv2.1/Kv8.2 channels to mediate transient hyperpolarizing overshoots of the membrane potential [79,80] and Kv8.2 mutations cause a cone dystrophy disorder [81]. Kv6.1, Kv8.1, and Kv8.2 have also been implicated in hyperexcitability of hippocampal neurons relevant to epilepsy [82,83,84].

KvS have also been implicated in chronic pain. Kv9.1 co-localises with Kv2.1 in myelinated sensory neurons that become hyperexcitable following nerve damage. Injury-induced Kv9.1 downregulation decreases Kv2.1 activity and enhances excitability, including spontaneous and evoked firing, and triggers pain hypersensitivity in rodents [85]. Consistent with this, deletion of the KCNS1 gene encoding Kv9.1 in mice results in basal and neuropathic pain sensitivity [86]. Kv2.1, but not Kv9.1, is also expressed in small nociceptors [74], but the composition of the native tetramers is not known. Since Kv2.1 conduction is sculpted by the modulatory influence of silent subunits, it is plausible that different Kv2.1/KvS combinations and stoichiometry can fine-tune excitability in distinct classes of sensory neurons. For example, Kv9.3 hyperpolarises the voltage dependence of Kv2 inactivation more substantially than Kv9.1 [78,87,88] and, even for a given KvS, the physiological impact is predicted to depend on whether firing is limited by the inactivation of inward currents [88]. The importance of Kv2.1 modulation by Kv9.1 in nociception is further underscored by the identification of two SNPs in the human Kv9.1 gene, which predisposes to the development of chronic pain [89]. Similarly, mutations in Kv6.4 may promote excitability of trigeminal neurons during migraine attacks [90] as well as pain during labor [91]. The role of KvS in pain is still poorly understood, and it may even include regulation of noncanonical Kv2.1 functions such as channel clustering and protein trafficking, as discussed below. Untangling this pathway could provide unique opportunities for pain treatments, which may prove advantageous compared to targeting the ubiquitously expressed Kv2.1 subunit.

A prime example of noncanonical Kv function is the formation of large clusters by Kv2.1 channels which localise to the neuronal membrane of the soma, proximal dendrites, and axon initial segment of CNS neurons [92]. In contrast to the active, diffused form of the channel, these micrometer-sized clusters are found to be primarily nonconductive [93,94] and are dispersed in response to neuronal activity, glutamate-induced excitotoxicity, hypoxia, or second messengers [95,96]. Regulation of cluster formation was originally thought to fine-tune neuronal excitability by dynamic control of the active vs. inactive forms but recent evidence hints towards a nonconducting role for Kv2 clusters. Thus, Kv2.1 and Kv2.2 clusters play a structural role in the formation of plasma membrane–endoplasmic reticulum junctions which serve as trafficking hubs for recruitment of several proteins (e.g., voltage-activated Ca^2+^ channels, VAMPs, AKAPs, kinases, and syntaxin), important for many neurophysiological processes like trafficking, neurotransmitter release, Ca^2+^ homeostasis, and burst firing [97,98,99,100,101]. Such a role is consistent with the identification of three Kv2.1 mutations which cause neurodevelopmental disorders despite the fact that they do not alter Kv2.1 conductance per se [102]. While Kv2 cluster formation has not been confirmed in peripheral sensory neurons, it is plausible that similar mechanisms operate in the pain-signaling pathways and that disruption of normal Kv2 clustering due to inflammation or injury affects nociceptive excitability.

Two P(ore) domain potassium channels (K2Ps) are known for facilitating a passive and rapid K^+^ flow at a range of membrane potentials. This leak (also called background) outward conductance stabilises resting membrane potential, assists repolarisation, and even enables AP generation in the absence of classical Kv channels [103]. Surprisingly, however, additional voltage-dependent activation has been documented in some K2P channels despite the absence of a canonical voltage-sensing domain with a positively charged S4-helix for gating by depolarisation [104,105]. Instead, voltage sensitivity appears to derive from movement of three or four ions into the high electric field of the selectivity filter [106], which then acts to “gate” the movement of K^+^ ions. Thus, in contrast to classical Kv channels where the properties of voltage sensing, activation, and inactivation can be mapped to distinct regions of the channel, K2Ps carry out these functions by employing different structural states of the selectivity filter. Moreover, many stimuli relevant to physiological functions such as PIP_2_, acidosis, and membrane stretch can switch off this voltage activation [107], limiting K2Ps to leak conductance by locking them open. Altogether, K2P channels are increasingly recognised as important modulators of polymodal pain perception. The best studied members TRAAK, TREK1, and TREK2 are mechano- and thermosensitive, albeit at different temperature ranges (TRAAK and TREK1, noxious temperatures; TREK2, moderate temperatures) [108,109,110,111,112]. Accordingly, deletion of these channels affects mechanical, heat, and oxaliplatin-induced cold sensitivity [108,109,112,113].

When covering atypical potassium channel function, a special mention should be made on inwardly rectifying potassium channels (Kir) which are mainly found in supporting cells such as glia. Kir channels are unique in that, at depolarised potentials, they preferentially mediate movement of K^+^ ions towards the inside of the cell in contrast to other potassium channels. The resulting inward currents help maintain resting membrane potentials and are therefore important in a number of physiological processes such as microglial activation during inflammation [114]. In addition, the buffering activity of Kir in nonneuronal cells prevents extracellular K^+^ accumulation, which would cause action potential “short-circuiting” and could detrimentally impact neuronal excitability [115,116]. Several members of the seven Kir subfamilies (Kir1–Kir7) have been specifically implicated in pain modulation. Kir4.1 channels expressed in satellite glia cells of the trigeminal ganglion appear to be important for facial pain. Silencing Kir4.1 expression in rats to mimic the effect of nerve injury or inflammation induces hyperexcitability and facial pain behaviours [117,118], while Kir4.1 knockout mice exhibit depolarised membrane potentials and inhibition of K^+^ uptake [119,120]. Members of the Kir3 family (also known as G protein-regulated inward rectifiers K^+^ channels, GIRK) are crucial mediators of spinal analgesia because their coupling to G proteins underlies analgesia conferred by endogenous and exogenous opioids [121,122]. Consistent with this, variations in the gene encoding GIRK2 are associated with pain phenotypes, as well as analgesic responses in humans [121,122,123]. Besides their established role in the CNS, GIRK2 expressed in sensory neurons may also contribute to peripheral antinociception induced by opioids [124].

Together, there is considerable interest in developing novel forms of analgesia by modulating K^+^ channel function, either correcting a primary pathology underpinning a pain state or nonspecifically reducing neuronal excitability. Much of the early interest focused on Kv channel openers, but more recent data suggests that drugs activating K2Ps may prove useful for a variety of pain symptoms [125]. Recently, a somewhat counterintuitive observation was that opening of ATP-sensitive potassium channels induces migraine attacks in migraineurs using a randomized, double-blind, placebo-controlled, crossover design [126]. The mechanism is not clear but might involve neuronal or nonneuronal processes, given the widespread distribution of these channels. It is worth noting that other K^+^ channels have a much more restricted distribution to nociceptive neurons.

## 4. TRP Channels

Transient receptor potential (TRP) channels form a group of 28 ion channels (27 in humans) organized into 7 families TRPC (canonical), TRPM (melastatin), TRPV (vanilloid), TRPA (ankyrin), TRPML (mucolipin), TRPP (polycystic), and TRPN (no mechanoreceptor potential C) [127,128]. Members of this family continue to be among the most studied in the ion channel field, in particular for pain as their relevance for certain pathophysiological conditions and peripheral sensory perception prompts them as targets for therapeutic modulation [129,130,131]. Canonical features of this family would be weak voltage-dependence [132], conductance for cations including divalent cations [133], and frequently, channel-specific rectification [134]. Below, some unexpected channel behavior is discussed.

TRPM3 has been found to generate an unexpected conductance when a combination of agonists was applied, namely pregnenolone sulfate and clotrimazole (or sole application of the agonist CIM0216) [135,136]. This ion permeation pathway, that allows inward rectification driven by Na^+^, has been likened to the omega pore in classical voltage-gated cation channels; however, the latter has been uncovered in disease-inducing mutations, whereas in the case of TRMP3, it exists in the wild-type channel. Moreover, in the case of clinically relevant clotrimazole plasma levels, it is feasible that, at 37 °C, circulating levels of pregnenolone sulfate can open this alternative pore [135]. Further analysis of the voltage-sensing domain of TRPM3 by means of site-directed mutagenesis revealed a critical role of several amino acids in the voltage-sensing domain for the formation of this alternative ion-permeation pathway [136]. In other channel families, including potassium, sodium, and proton channels, channel mutations have also been shown to cause noncanonical pores, as discussed above [51,137,138]. Ultimately, such unexpected behaviour can contribute to a larger goal, understanding the gating and overall ion channel molecular mechanics.

The majority of TRP channels is outwardly rectifying, despite some exceptions such as TRPML1/2/3 [139] and TRPV5/6 [140]. In case the rectification is not dependent on divalent cations, causing asymmetry by an open channel block [141,142], this is an intrinsic property of the channel. This property can be changed by a single helix-breaking amino acid, as shown for the TRPML3-A419P mutation [143].

Increasing response to continuous agonist exposure: TRPA1 stands out by being most sensitive to modification by electrophilic molecules, a feature which critically involves cysteine residues on the N terminus of the channel [144,145,146,147]. Irrespective of the mode of activation, a dilation of the pore has been described for TRPA1, a feature attributed only to a few ion channels, including TRPV1 [148]. This pore dilation has a time constant below 10 s [149,150]. It should be mentioned that an alternative explanation to dilations of the pore has been proposed [151] (summarized in Reference [152]).

However, a slow but several-fold increase in conductance upon continuous agonist exposure with a much longer time course has been demonstrated [153]. These current increases can be better studied in the absence of calcium, as calcium influx causes a calcium-dependent desensitisation, and both mechanisms seem to balance each other. This allows continuous activation through TRPA1, where other channels show extensive tachyphylaxis or desensitisation. The mechanism is not PKA- or PKC-dependent. The topic has been further investigated using the noncovalent agonist carvacrol [154]. In contrast to the slow covalent action of TRPA1 by allyl isothiocyanate, the time constant for activation by carvacrol was 3.1 s, which allowed for tracking the current faster. A similar agonist-induced current increase was detected, and the current observed after a previous exposure is picked up almost invariable to the period between stimuli. The time constant of agonist-induced sensitisation was 130 s, which is well above all other described processes. Agonist exposure is required for this effect, as it could not be reproduced by opening the channel using voltage stimulation. Agonist-induced sensitisation occurred between covalent and non-covalent agonists, indicating a modification which is common to all agonists but upstream or independent of voltage-induced gating. However, a current through the channel was not required, as the exposure time-dependent current increase progresses when flux is inhibited by the additional presence of an antagonist. Similar to allyl isothiocyanate, a desensitisation was observed for saturating concentrations of carvacrol; the reason for this remaining unclear. The agonist-induced sensitisation was assumed to bring TRPA1 into a hypothesized state, which has a far left-shifted voltage dependence [154]. Inhibition of ATP-dependent mechanisms and membrane trafficking also did not affect the observation. TRPA1 has been investigated using long exposures mainly due to the slow onset required for the covalent agonists. This is not required for other channels; therefore, such protocols might simply not have been tested so far. It should be mentioned that a shift in concentration-dependent binding with prolonged agonist exposure has been reported in other receptors [155]; an “imprinting” by a lasting conformational change was hypothesized. For TRPA1, the change in receptor binding after prolonged exposure has not been investigated.

## 5. HCN Channels

Hyperpolarization-activated cyclic nucleotide-gated (HCN) channels comprise a small family with four members, HCN1–HCN4 [156,157,158,159,160]. The channels are related to CNG and Kv channels [161,162,163] but are distinguished by several unique features. HCN channels are controlled by the membrane potential; however, in contrast to most other voltage-gated ion channels, hyperpolarization but not depolarization opens them. Second, the channels contain the GYG motif in the pore region, which constitutes the potassium selectivity filter in potassium channels. Nevertheless, HCN channels are nonselective cation channels conducting both sodium and potassium ions (selectivity for K^+^/Na^+^ ~4:1). Under physiological conditions, activation of the channels leads to influx of sodium ions, resulting in depolarization. Third, cyclic nucleotides, particularly cAMP, stimulate the channels by accelerating their activation kinetics and by shifting the activation curve in the positive direction. However, cAMP is not required for channel opening. HCN channels contain a cyclic nucleotide-binding domain (CNBD) in the carboxyterminus. Truncation experiments have shown that the cyclic nucleotide-binding domain inhibits gating in the cAMP-unbound state, whereas cAMP binding relieves this inhibition [164].

The recent cryo-EM structure of HCN1 [165] together with the crystal structure of a cyclic nucleotide-binding domain [166] yielded important insights into the peculiar characteristic outlined above. As compared to potassium channels, the outer half of the selectivity filter in HCNs is enlarged and two of the potential four potassium binding sites are lacking. This results in a loss of the kinetic selectivity for potassium present in potassium channels, where four binding sites in the permeation pathway are present. Second, the closed pore is stabilized by the voltage sensor (S4 segment) and other domains in the depolarized state. It is proposed that the hyperpolarization-induced downward movement of S4 disrupts these interactions, leading to spontaneous pore opening. Third, binding of cAMP induces small conformational changes, leading to a rotation towards opening of the inner gate. However, HCN1 is barely modulated by cyclic nucleotides, so it remains to be seen if this structural mechanism also operates in HCN2 and HCN4, which are strongly modulated by cAMP.

The individual isoforms possess characteristic properties, which have been investigated in heterologous expression systems and were confirmed by using knockout mice of each isoform [167,168,169,170,171]. Beneath the different sensitivities toward cyclic nucleotides, the isoforms strongly differ in the rate of channel opening. HCN1 is the fastest activating isoform, HCN2 and HCN3 possess an intermediate activation kinetic, and HCN4 is the slowest HCN channel with an activation time constant up to several seconds.

In principle, activation of HCN channels leads to depolarisation and promotes AP generation. Since neuronal hyperexcitability and spontaneous AP generation of nociceptors contribute to the generation of pathological pain, HCN channels and, in particular, HCN2 may be involved in the sensitization of nociceptors in chronic pain conditions. In line with this assumption, an enhancement of the current flowing through these channels (I_h_) has been directly shown in different models of neuropathic [172,173,174] and inflammatory [175] pain. The increase in I_h_ has been attributed to an upregulation of HCN transcript and/or protein [113,175,176,177,178,179], upregulation of the potential auxiliary subunit MiRP1 [174], increased intracellular cAMP levels [180], and PKA-dependent phosphorylation of HCN2 [181]. Nociceptor-specific deletion of HCN2 by using a Nav1.8-Cre transgene to delete the floxed HCN2 exons directly demonstrated the important role of this channel in pathological pain conditions [179,182]. In two different models of neuropathic pain, HCN2 emerged as a key regulator since its deletion strongly reduced [183] and even abolished [182] the increase in nociceptive sensitivity. Moreover, in diabetic mice, deletion or block of HCN2 prevented the mechanical allodynia following diabetic neuropathy [180]. In inflammatory pain, the importance of HCN2 was also shown, but the extent differed between the inflammatory compound (carrageenan, PGE2, 8-bromo-cAMP, zymosan A, and CFA) and behavioural test (mechanical and heat hypersensitivity) used [179,182]. It is proposed that HCN2 channels determine nociceptor hypersensitivity if the inflammatory signal transduction pathways result in an increase of cAMP, which may directly modulate channel activity via binding to the CNBD [184] or indirectly via activation of PKA and phosphorylation of HCN2 or associated proteins [183].

However, in spite of these promising findings in murine models, a recent human phase 2 study did not find any effect of ivabradine on capsaicin-induced hyperalgesia and pain in healthy volunteers [185]. Ivabradine caused a significant heart-rate reduction, indicating that the dose was sufficient to block HCN4 and HCN1 channels in the sinoatrial node. These results suggest that it might be necessary to develop HCN2-selective substances (which do not cross the blood–brain barrier [167]) to serve as analgesics. Beyond that, it is still possible that ivabradine is effective in other human pain models distinct from the neurogenic inflammation induced by TRPV1 activation.

## 6. Conclusion

Unexpected properties of several ion channels with importance for the pain field were discussed (Table 1). We hope that the selective and non-comprehensive choices help to transfer knowledge within the field. Considering the possibility that such findings in other channels might explain otherwise not understood issues and facilitate scientific progress.

## Figures and Tables

**Table 1:** Overview for the mentioned noncanonical ion channel features: canonical features not discussed in the review are considered ligand- and voltage-gating, selective permeability, interaction with other proteins, fractional presence at the plasma membrane, heteromerisation, modulation by intracellular cascades, and a change of expression.

Noncanonical Property	Ion Channel and References
slow inactivation	**Nav** [9,12,13,14,15,16,17,18,19,20,21,22,23,24,25,26,27,28,29,30,31,32,33]
resurgent current during membrane repolarisation	**Nav (1.2, 1.5, 1.6, 1.7, 1.8)** [29,34,35,36,37,38,39,40,41]
side fenestrations of the channel pore	**Nav** [9,10,46,47,48,49]
alternative ion permeation pathway across the membrane	**Nav (1.2, 1.4, 1.5)** [50,51,52,53,54,55] **TRPM3** [135,136,137,138]
electromechanical coupling of transmembrane domain S4 and S5 residues, facilitating “rack-and-pinion” movements	**Kv** [62,63,64]
mechanically induced current	**Kv (1.1)** [65,66]
susceptibility to epigenetic silencing	**Kv (1.2)** [67,68,69,70,71]
action potential frequency filtering by silent subunits	**Kv (2.1)–KvS (members of the Kv5, 6, 8, 9 families)** [72,73,74,75,76,77,78,79,80,81,82,83,84,85,86,87,88]
formation of channel clusters	**Kv (2.1, 2.2)** [92,93,94,95,96,97,98,99,100,101]
voltage-dependent activation in the absence of a canonical voltage-sensing domain	**K2P** [104,105,106,107]
inward currents at depolarizing potentials	**Kir** [114,115,116,117,118,119,120] **TRPML1-3** [139,141,142,143] **TRPV5/6** [140]
pore dilation	**TRPA1** [149,150,152] **TRPV1** [148]
sensitization due to continuous agonist exposure	**TRPA1** [32,147,149,150,151,152,153,154]
open probability increases upon hyperpolarization	**HCN 1–4** [156,157,158,159,160,161,162,163]
nonselective cation channel despite a typical potassium selectivity filter motif	**HCN 1–4** [156,157,158,159,160,161,162,163]

## Abbreviations

AKAP	A-kinase anchor protein
HCN	Hyperpolarization-activated cyclic nucleotide-gated channel
Kv	voltage-gated potassium channel
KvS	Silent potassium channel subunit
K2P	Two Pore domain potassium channel
Kir	Kir inwardly rectifying potassium channel
Nav	Voltage-gated sodium channel
TRP	Transient receptor potential channels
VAMP	Vesicle-associated membrane protein
